# Supplementary material for: Cell signaling pathways discovery from multi-modal data
Source: bioRxiv. 2025 Feb 8:2025.02.06.636961. Preprint. [Version 1] doi: 10.1101/2025.02.06.636961 (PMC11839107; doi:10.1101/2025.02.06.636961)
Supplement: Supplement 1 [file media-1.pdf]

Supplementary materials to
Cell signaling pathways discovery from multi-modal data

Changhan He[1]*, Claire Simpson[2]*, Ian Cossentino[2], Bin Zhang[2], Sasha Tkachev[2], Devon J. Eddins[3], Astrid Kosters[3], Junkai Yang[3], Shivani Sheth[2], Tyler Levy[2], Anthony Possemato[2], Linglin Huang[4,5], Evgeniy Tabatsky[6], Ivan Gregoretti[2], Majd Ariss[2], Deepti Dandekar[7], Aniket Ausekar[7], Eliver E. B. Ghosn[3], Marco Colonna[8], Klarisa Rikova[2], Qing Nie[1#], Darya Orlova[2#]

[1] Department of Mathematics, University of California, Irvine, California, 92697, USA

[2] Cell Signaling Technology, Danvers, Massachusetts, 01915, USA

[3] Division of Immunology and Rheumatology, Department of Medicine, Lowance Center for Human Immunology, Emory University School of Medicine, Atlanta, Georgia, 30322, USA

[4] The Gene Lay Institute of Immunology and Inflammation, Brigham and Women's Hospital, Massachusetts General Hospital and Harvard Medical School, Boston, Massachusetts, 02115, USA

[5] Klarman Cell Observatory, Broad Institute of MIT and Harvard, Cambridge, Massachusetts, 02142, USA

[6] Independent researcher, Komsomolsk-on-Amur, 681021, Russia

[7] Evolvus Technologies Pvt. Ltd., Pune, Maharashtra 411030, India

[8] Department of Pathology and Immunology, Washington University School of Medicine, St. Louis, Missouri, 63110, USA

# To whom correspondence should be addressed: qnie@uci.edu; dyorlova@gmail.com

* CH and CS are joint first authors

This PDF file includes:

Materials and Methods
Supplementary Figures 1 to 8
Supplementary Tables 1 to 2
Captions for Supplementary Data 1 to 3

Other Supplementary Materials for this manuscript include the following:

Supplementary Data 1. Benchmarking table comparing the ability of GSEA, exFINDER, and Incytr to rediscover known cancer-specific, 5XFAD model-specific, and COVID-specific pathways.

Supplementary Data 2. A list of references supporting the primary pairwise L-R, R-EM, and EM-T interactions presented in this paper (Figures 3-5).

Supplementary Data 3. Output of the Incytr analysis on the 5XFAD dataset, focusing on interactions involving Apoe, App, Trem2, and Lrp1.

Materials and Methods

Data/Sample Overview and Analysis

MC38 Dataset

Generation of the MC38 Mouse Model

Cell Culture

The MC38 murine colon adenocarcinoma cell line was obtained from Kerafast and cultured according to standard protocols. Cells were maintained in high-glucose Dulbecco's Modified Eagle Medium (DMEM; Gibco, Thermo Fisher Scientific) supplemented with 10% heat-inactivated fetal bovine serum (FBS; Gibco), 1% penicillin-streptomycin (Pen/Strep; Gibco), and 2 mM L-glutamine at 37°C in a humidified atmosphere containing 5% CO_2.

Preparation of Cells for Injection

MC38 cells were harvested during the logarithmic growth phase to ensure high viability and proliferation rates. Cells were detached using 0.25% trypsin-EDTA (Gibco) and resuspended in sterile phosphate-buffered saline (PBS; pH 7.4). Cell concentration and viability were assessed using a hemocytometer and trypan blue exclusion method, respectively. Cell suspensions were adjusted to a final concentration of 1×10^6 cells per 100 µL of PBS for injection.

Animal Handling and Ethical Approval

All animal procedures were conducted in compliance with the Institutional Animal Care and Use Committee (IACUC) guidelines of Bluefin Biomedicine. Female C57BL/6 mice, aged 6–8 weeks, were purchased from The Jackson Laboratory and housed under specific pathogen-free conditions with a 12-hour light/dark cycle. Mice were allowed to acclimate for one week prior to experimentation and had ad libitum access to food and water.

Subcutaneous Tumor Implantation

For tumor induction, mice were briefly anesthetized with isoflurane inhalation (induction at 3–4%, maintenance at 1–2%). Using a 27-gauge needle, 0.5×10^6 MC38 cells in 100 µL of PBS were injected subcutaneously into the right flank of each mouse. Mice were monitored until full recovery from anesthesia and then returned to their cages.

Tumor Monitoring

Tumor growth was measured three times per week using digital calipers. Tumor volume was calculated using the formula:

Tumor volume = 1/2(*length* × *width*^2)

where length is the longest dimension and width is the dimension perpendicular to the length. Mice were also monitored for signs of discomfort, weight loss, or any adverse effects. Humane endpoints were established, and mice were euthanized if tumor volume exceeded 1,500 mm^3 or if they exhibited signs of distress.

MC38 scRNAseq Data Generation and Analysis

MC38 tumor samples were dissociated using a GentleMACs, and mouse tumor dissociation kit (Miltenyi Biotec). Cell suspensions were subsequently labeled using Biolegend’s TotalSeq-A anti-mouse hashtag antibodies. Single cell suspensions were counted, pooled, and analyzed following 10X Genomics Single Cell 3' v2 assay. Resulting libraries were analyzed on an Illumina NovaSeq SP 100-cycle run. The resulting sequencing data was analyzed using CellRanger 3.1.0, and searched with mm10-3.0.0 reference.

Seurat was used to perform quality control, normalization, scaling, dimensionality reduction, and clustering. Marker genes were used to annotate cell types. To distinguish between EMT cells and other cell types we relied on previously reported EMT signatures [Taube et al., 2010; Anastassiou et al., 2011; Tian et al., 2020; Gilles et al., 2013]. In particular, Dcn, Serpinh1, Mmp9, Serpinf1, Zeb1 and Col6a2 genes were found to be upregulated in EMT cells (as well as some fibroblasts) and not in cancer cells.

MC38 Proteomics Data Generation

Lysis and Protein Extraction

Flash frozen samples were resuspended and probe tip sonicated in 8M urea with 200mM HEPEs pH8.5 with 1x Protease and Phosphatase inhibitors (CST #5872). Samples were then reduced for 30 minutes at 50C with 5mM DTT. Samples were alkylated in the dark for 30 minutes at room temperature with 10mM iodoacetamide. Alkylation was quenched with 5 mM DTT.

Protein Digestion

Samples were diluted to 2M urea with 20mM HEPEs pH 8.5 with 1mM CaCl2, and digested overnight at 37C with LysC (Wako-Chem). Samples were further diluted to 1M urea with 20 mM HEPEs pH 8.5 with 1mM CaCl2 and digested for 6 hours with Trypsin (Pierce). Samples were acidified with trifluoroacetic acid (TFA) de-salted with a Waters SepPak C18 column. Peptide concentrations were estimated with a microBCA kit from Pierce (#23235). Peptides were TMT-labeled in 200 mM HEPEs pH 8.5 with 30% acetonitrile for 1 hour, combined, dried in a speedvac, and desalted with a SepPak C18 cartridge.

Total Protein Analysis Sample Preparation

For total protein analysis, 100 ug of combined peptide material was resuspended in buffer A (10mM NH 4 HCO 2, pH10, 5% ACN), and fractionated on a Zorbax Extended C18 column (2.1 × 150 mm, 3.5 μm, no. 763750-902, Agilent) using a gradient of 10-40% bRP buffer B (10 mM NH 4 HCO 2, pH10, 90% ACN). 96 fractions were collected and concatenated into 24 fractions. Fractions were dried, de-salted with stop-and-go extraction tips (STAGE-tips), and dried for LC-MS analysis.

Enrichment of Post-translationally Modified Peptides

3mgs of combined labeled peptides were IMAC enriched using High-Select TM Fe-NTA Phosphopeptide Enrichment Kits (Thermo Fisher Scientific, San Jose, CA). The remainder of the labeled pool of peptides was analyzed using PTMScan® HS

Phospho-Tyrosine (P-Tyr-1000) Kit #38572 from Cell Signaling Technologies. The resulting phosphopeptide elution from IMAC enrichment was fractionated as described above, using a gradient from 5-40% Buffer B. 96 fractions were collected and concatenated into 12 fractions. Fractions were dried, de-salted with STAGE-tips, and dried for LC-MS analysis.

LC–MS Analysis of Total Protein

Samples were analyzed on an Orbitrap Fusion Lumos mass spectrometer (Thermo Fisher Scientific, San Jose, CA) coupled with a Proxeon EASY-nLC 1200 liquid chromatography (LC) pump (Thermo Fisher Scientific, San Jose, CA). Peptides were separated on a 100 μm inner diameter microcapillary column packed with ~40 cm of Accucore150 resin (2.6 μm, 150 Å, ThermoFisher Scientific, San Jose, CA). For each analysis, we loaded approximately 1 μg onto the column. Peptides were separated using either a 2.5 h gradient of 6–30% acetonitrile in 0.1% formic acid with a flow rate of 550 nL/min. Each analysis used an SPS-MS3-based TMT method [Ting et al., 2011; McAlister et al., 2014] , which has been shown to reduce ion interference compared to MS2 quantification [Paulo et al., 2016]. The scan sequence began with an MS1 spectrum (Orbitrap analysis, resolution 120,000; 350-1400 m/z, automatic gain control (AGC) target $4.0 \times 10^{\wedge 5}$, maximum injection time 50 ms). Precursors for MS2/MS3 analysis were selected using a Top10 method. MS2 analysis consisted of collision-induced dissociation (quadrupole ion trap; AGC $2.0 \times 10^{\wedge 4}$; normalized collision energy (NCE) 35; maximum injection time 22 ms). Following acquisition of each MS2 spectrum, we collected an MS3 spectrum, a method in which multiple MS2 fragment ions are captured in the MS3 precursor population using isolation waveforms with multiple frequency notches [McAlister et al., 2014]. MS3 precursors were fragmented by HCD and analyzed using the Orbitrap (NCE 65, AGC $3.5 \times 10^{\wedge 5}$, maximum injection time 120 ms, isolation window 1.2 Th, resolution was 50,000 at 200 Th).

LC–MS Analysis of PTM Enriched Fractions

For the analysis of PTM-enriched samples, the same MS and HPLC instruments were used as described above. For each analysis, approximately 500 ngs of enriched

peptides were loaded on the column and run over a 120-minute gradient of 2-32% acetonitrile in 0.1% formic acid with a flow rate of 400 nL/min. MS1 spectra were collected in the Orbitrap at a resolution of 60,000 with a scan range of 300-1500 m/z using an AGC target of 4.0e5 with a maximum injection time of 25ms. Peptides for MS2 analysis were isolated using the quadrupole with an isolation window of 0.8 m/z. MS2 spectra were generated using Higher-energy collision dissociation (HCD) with a collision energy of 40%. Fragments were collected in the Orbitrap at a resolution of 50,000 with a first mass of 110 m/z, an AGC target of 5.0e4, and a maximum injection time of 200ms.

5XFAD Dataset

5XFAD snRNAseq Data

We used previously published snRNA data from 5XFAD and WT mouse brains (n = 3) [Zhou et al., 2020]. Seurat was used to perform quality control, normalization, scaling, dimensionality reduction, and clustering. Cell types were identified according to the expression of marker genes selected in the original study.

5XFAD Proteomics Data Generation

One male and one female half mouse brains from WT and 5XFAD backgrounds were analyzed. Samples were additional replicates from the study from which the matched snRNAseq data were generated [Zhou et al., 2020]. Total and phosphoproteome datasets were generated and analyzed as described above for the MC38 tumor samples with the following exceptions. After sample lysis, protein concentration was estimated using microBCA kit from Pierce. 1 mg of protein per sample (in triplicate) was then reduced, and alkylated as described above. Proteins were then precipitated using a MeOH Chloroform extraction. Protein pellets were resuspended in 200 uL of 200 mM EPPS buffer (pH 8.5), digested with 10µg of LysC overnight at 37C, and 10 µg of Trypsin was added for an additional 6-hour digestion. Peptides were then TMT labeled in 200mM EPPS with 30% AcN for 1 hour at room temperature using TMT-pro reagents from Thermo.

Bulk Total Proteomics Data Analysis

Mass Spectra were processed using a Comet-base software pipeline [Eng et al., 2012; Huttlin et al., 2010]. Resulting data were searched with a fully tryptic database containing mouse Swissprot consensus entries plus isoforms allowing for a static modification of lysine and N-termini with TMT (229.1629 Da) or TMT-pro (304.2071 Da) and carbamidomethylation (57.0215 Da) of cysteine, along with variable oxidation (15.9949 Da) of methionine. Searches were performed using a 20 ppm precursor ion tolerance, the production tolerance was set to 1.0 Th. Peptide-spectrum matches (PSMs) were adjusted to a 2% false discovery rate (FDR) using previously described linear discriminant analysis [Elias & Gygi, 2007; Elias & Gygi, 2009]. Filtered PSMs were collapsed to a final protein-level FDR of < 2%. Protein assembly was guided by principles of parsimony to produce the smallest set of proteins necessary to account for all observed peptides [Huttlin et al., 2010]. MS3 spectra with TMT reporter ion summed signal-to-noise ratios less than 100 were excluded from quantitation [McAlister et al., 2012].

Bulk Phosphorylation MS Data Analysis

PTM data was analyzed as described above, with a fragment ion tolerance of 0.02 Th instead. Variable modifications were considered for phosphorylation on serine, threonine, and tyrosine (79.9663 Da). PTM sites with A-score values > 13 were considered as localized for downstream analysis [Huttlin et al., 2010; Beausoleil et al., 2006].

Kinase-Substrate Matching

Phosphorylation sites within provided phosphoproteomic datasets (MC38 and 5XFAD) were analyzed for associated kinases using previously described methods [Johnson et al., 2023]. Briefly, 10-mer segments surrounding S/T/Y residues were compared against experimentally determined sequence motifs representing each kinase’s most biochemically favorable substrate and assigned a score. Kinases were then ranked according to the percentile of that site’s score with respect to scores for all sites in the phosphoproteome for that kinase. Kinase-substrate matching is performed by

determining the (15 for phosphoY, 5 for pS/pT) most highly ranked kinases for a given site. Site scoring is publicly available at https://kinase-library.phosphosite.org/.

COVID-19 Dataset

There are two cohorts of healthy control (HC) and COVID-19 patient samples that were used in this study. The first cohort (six HC and four COVID-19 patient samples previously sequenced) and a second cohort (three HC and three COVID-19 patient samples not previously sequenced) collected as part of the previously published report [Eddins et al., 2023] were processed as part of this study. For metadata associated with these patients see Supplemental Table 1 from Eddins et al., 2023 [Eddins et al., 2023]. The first cohort patient IDs: PHA1005, PHA1006, PHA1007, PHA1008, PHA1009, PHA1010, PHA0007, PHA0008, PHA0010, PHA0018; the second cohort patient IDs: PHA1005, PHA1007, PHA1008, PHA0001, PHA0002, PHA0003.

Whole blood samples from consenting donors were collected and processed under institutional review board (IRB)-approved protocols as previously described [Eddins et al., 2023]. Following plasma isolation, red blood cells (RBCs) were depleted from whole blood using the EasySep™ RBC Depletion Reagent (STEMCELL Technologies). Following RBC-depletion, cells for scRNA-seq were surface stained with a panel of 89 antibody-derived tags (ADT; see Supplemental Table 4 in [Eddins et al., 2023]) and loaded to target encapsulation of 10,000 cells using a Chromium Controller (10X Genomics, Pleasanton, CA). Gene expression (GEX) and ADT libraries were generated using the Chromium Single Cell 5′ Library & Gel Bead Kit v1.1 with feature barcoding protocol per the manufacturer's instructions and sequenced in a S4 flow cell on a NovaSeq 6000 (Illumina, San Diego, CA). Peripheral blood mononuclear cells (PBMCs) were isolated using either the EasySep PBMC isolation kit or Lymphoprep (StemCell Technologies), from the remaining cells and were frozen at -196˚C in 10% ultra-pure grade (≥99.9%) dimethyl sulfoxide (DMSO; VWR) in fetal bovine serum (FBS, Thermo Fisher Scientific) for future analyses.

InTraSeq Experiment

Frozen PBMCs from Healthy and COVID-19 infected donors from the second cohort were obtained from the Ghosn lab. The cells were washed once with 10 mL of Roswell Park Memorial Institute (RPMI)-1640 medium then spun down at 300 x g for 5 minutes at 4°C. The cells were washed again with 10 mL of Phosphate-Buffered Saline (PBS), spun down at 300 x g for 5 minutes at 4°C, then were resuspended in 500 uL of PBS prior to be subjected to the InTraSeq protocol (CST #82906) [Ariss et al., 2024]. The Following conjugates were added in the Immunostaining step: InTraSeq™ 3' Conjugate Antibody Cocktail 1 (CST #48167), S100A9 (D5O6O) Rabbit mAb (InTraSeq™ 3' Conjugate 3004) #97056, T-bet/TBX21 (D6N8B) XP® Rabbit mAb (InTraSeq™ 3' Conjugate 3009) #57412, CD68 (D4B9C) XP® Rabbit mAb (InTraSeq™ 3' Conjugate 3012) #29105, CD3 (UCHT1) Mouse mAb (InTraSeq™ 3' Conjugate 3028) #72489, CD4 (RPA-T4) Mouse mAb (InTraSeq™ 3' Conjugate 3029) #87589, CD8α (SK1) Mouse mAb (InTraSeq™ 3' Conjugate 3030) #25292, Mouse (G3A1) mAb IgG1 Isotype Control (InTraSeq™ 3' Conjugate 3001) #59605.

10x Genomics and Illumina Sequencing

Following InTraSeq, the cells in the second cohort of the covid dataset were processed using the 10x Genomics Next-Gem 3’ single cell kits and User Guide CG000317. After the single cell RNA and protein library preparation were generated, the samples were pooled and sequenced using the NextSeq 2000 and the P2 or P3 kits (100 cycles). Base-calling was performed using NextSeq 1000/2000 Control Software v1.5.0 (RTA v3.10.30).

Single Cell InTraSeq Analysis

InTraSeq data were loaded into a Seurat object along with the RNAseq data. Cells were filtered on the number of features (500-3000) and counts (900-7000) in the RNA data, the number of counts (<10000) in the protein data, and the percentage of mitochondrial genes (<5%). The protein data was normalized using centered log ratio transformation. Proteins were visualized using a minimum cutoff of quantile5 or quantile10 and a maximum cutoff of quantile90 or quantile95 expression.

Mapping Incytr-identified Pathways onto WikiPathways

To interrogate and gain more insight into the biological significance of all the paths predicted by Incytr, we downloaded and mapped these four-step paths onto WikiPathways gene sets [Agrawal et al., 2024]. These gene sets are derived from the WikiPathways biological pathway database (https://www.wikipathways.org/) and downloaded from the Molecular Signatures Database (MSigDB) (https://www.gsea-msigdb.org/gsea/msigdb) [Liberzon et al., 2011] curated gene set collections, specifically the canonical pathway subcollections. There are 830 human and 189 mouse gene sets/pathways, which are canonical representations of signaling pathways compiled by domain experts. We developed a Python script that overlays the four components of Incytr paths onto WikiPathways. To extend the coverage, we converted mouse gene names to their human equivalents. The Python script is provided and can be downloaded from our software package.

Liceptor, Biomarker and Protein Degraders Databases

The Liceptor database (https://www.evolvus.com/data/liceptor-database) is a gold standard in small molecule drug discovery. With over 10 million compounds, it is the world's most comprehensive and largest small-molecule ligand bioactivity dataset built by extracting and curating data from patents drafted in English, Japanese, Chinese, and Korean. It includes 2D structures, associated molecular descriptors, and bioactivity data such as assays, functions, and therapeutic indications. The database focuses on various target families - GPCRs, ion channels, transporters, kinases, proteases, phosphatases, nuclear receptors, cytochrome P450 covering over 19000 targets.

The Biomarker database (https://www.evolvus.com/data/biomarker-database) is a database of Oncology Biomarkers, a curated database of genomic/molecular alterations, investigational and approved therapies and clinical trials. This curated database includes, the various biological, chemical and clinical data points, including but not limited to other properties of the therapies against the biomarkers, if present. The information is derived from different sources such as – trial registries, regulatory filings, labels.

Proximers - The Protein Degraders database (https://evolvus.com/data/proximers-database) is the world's largest curated resource that compiles information on bi-functional and hetero bi-functional compounds, along with their associated bioactivity and pharmacokinetic metadata. The data is sourced from various sources, including patents drafted in English and non-English languages.

The database includes chemical and biological data points related to the following components: protein binding moiety, ubiquitin ligase binding moiety, linker, protein degrader.

Incytr-discovered Pathway Embeddings

An aggregation of Incytr-derived four-step signaling pathways was used to train the Doc2Vec model (https://radimrehurek.com/gensim/models/doc2vec.html), independently for each dataset (~1M pathways for MC38, ~96k for 5XFAD, and ~116k for COVID) with the following input parameters: vector_size=60, window=2, min_count=1, epochs=10. All gene names were converted to lowercase to prevent artificial heterogeneity between mouse and human genes, as human gene names are typically written in all capital letters. After training, the embeddings were extracted and concatenated with the quantitative information related to the adjusted log2 fold change value (aFC value) calculated for each pathway using signaling probabilities $P_{i,j}^{k}$ between conditions (e.g., 5XFAD vs WT). These concatenated data were visualized using UMAP (https://umap-learn.readthedocs.io/en/latest/), with default settings (n_neighbors=5, random_state=42).

References

Taube, J. H., Herschkowitz, J. I., Komurov, K., Zhou, A. Y., Gupta, S., Yang, J., Hartwell, K., Onder, T. T., Gupta, P. B., Evans, K. W., Hollier, B. G., Ram, P. T., Lander, E. S., Rosen, J. M., Weinberg, R. A., & Mani, S. A. (2010). Core epithelial-to-mesenchymal transition interactome gene-expression signature is associated with claudin-low and metaplastic breast cancer subtypes. In Proceedings of the National Academy of Sciences (Vol. 107, Issue 35, pp. 15449–15454). Proceedings of the National Academy of Sciences. https://doi.org/10.1073/pnas.1004900107

Anastassiou, D., Rumjantseva, V., Cheng, W., Huang, J., Canoll, P. D., Yamashiro, D. J., & Kandel, J. J. (2011). Human cancer cells express Slug-based epithelial-mesenchymal transition gene expression signature obtained in vivo. In BMC Cancer (Vol. 11, Issue 1). Springer Science and Business Media LLC. https://doi.org/10.1186/1471-2407-11-529

Tian, S., Peng, P., Li, J., Deng, H., Zhan, N., Zeng, Z., & Dong, W. (2020). SERPINH1 regulates EMT and gastric cancer metastasis via the Wnt/β-catenin signaling pathway. In Aging (Vol. 12, Issue 4, pp. 3574–3593). Impact Journals, LLC. https://doi.org/10.18632/aging.102831

Gilles, C., Newgreen, D. F., Sato, H., & Thompson, E. W. (2013). Matrix Metalloproteases and Epithelial-to-Mesenchymal Transition: Implications for Carcinoma Metastasis. In Madame Curie Bioscience Database. Landes Bioscience. https://www.ncbi.nlm.nih.gov/books/NBK6387/

Ting, L., Rad, R., Gygi, S. P., & Haas, W. (2011). MS3 eliminates ratio distortion in isobaric multiplexed quantitative proteomics. In Nature Methods (Vol. 8, Issue 11, pp. 937–940). Springer Science and Business Media LLC. https://doi.org/10.1038/nmeth.1714

McAlister, G. C., Nusinow, D. P., Jedrychowski, M. P., Wühr, M., Huttlin, E. L., Erickson, B. K., Rad, R., Haas, W., & Gygi, S. P. (2014). MultiNotch MS3 Enables Accurate, Sensitive, and Multiplexed Detection of Differential Expression across Cancer Cell Line Proteomes. In Analytical Chemistry (Vol. 86, Issue 14, pp. 7150–7158). American Chemical Society (ACS). https://doi.org/10.1021/ac502040v

Paulo, J. A., O'Connell, J. D., & Gygi, S. P. (2016). A Triple Knockout (TKO) Proteomics Standard for Diagnosing Ion Interference in Isobaric Labeling Experiments. In Journal of

the American Society for Mass Spectrometry (Vol. 27, Issue 10, pp. 1620–1625). American Chemical Society (ACS). https://doi.org/10.1007/s13361-016-1434-9

Zhou, Y., Song, W. M., Andhey, P. S., Swain, A., Levy, T., Miller, K. R., Poliani, P. L., Cominelli, M., Grover, S., Gilfillan, S., Cella, M., Ulland, T. K., Zaitsev, K., Miyashita, A., Ikeuchi, T., Sainouchi, M., Kakita, A., Bennett, D. A., Schneider, J. A., … Colonna, M. (2020). Human and mouse single-nucleus transcriptomics reveal TREM2-dependent and TREM2-independent cellular responses in Alzheimer's disease. In Nature Medicine (Vol. 26, Issue 1, pp. 131–142). Springer Science and Business Media LLC. https://doi.org/10.1038/s41591-019-0695-9

Eng, J. K., Jahan, T. A., & Hoopmann, M. R. (2012). Comet: An open‐source MS/MS sequence database search tool. In PROTEOMICS (Vol. 13, Issue 1, pp. 22–24). Wiley. https://doi.org/10.1002/pmic.201200439

Huttlin, E. L., Jedrychowski, M. P., Elias, J. E., Goswami, T., Rad, R., Beausoleil, S. A., Villén, J., Haas, W., Sowa, M. E., & Gygi, S. P. (2010). A Tissue-Specific Atlas of Mouse Protein Phosphorylation and Expression. In Cell (Vol. 143, Issue 7, pp. 1174–1189). Elsevier BV. https://doi.org/10.1016/j.cell.2010.12.001

Elias, J. E., & Gygi, S. P. (2007). Target-decoy search strategy for increased confidence in large-scale protein identifications by mass spectrometry. In Nature Methods (Vol. 4, Issue 3, pp. 207–214). Springer Science and Business Media LLC. https://doi.org/10.1038/nmeth1019

Elias, J. E., & Gygi, S. P. (2009). Target-Decoy Search Strategy for Mass Spectrometry-Based Proteomics. In Methods in Molecular Biology (pp. 55–71). Humana Press. https://doi.org/10.1007/978-1-60761-444-9_5

McAlister, G. C., Huttlin, E. L., Haas, W., Ting, L., Jedrychowski, M. P., Rogers, J. C., Kuhn, K., Pike, I., Grothe, R. A., Blethrow, J. D., & Gygi, S. P. (2012). Increasing the Multiplexing Capacity of TMTs Using Reporter Ion Isotopologues with Isobaric Masses. In Analytical Chemistry (Vol. 84, Issue 17, pp. 7469–7478). American Chemical Society (ACS). https://doi.org/10.1021/ac301572t

Beausoleil, S. A., Villén, J., Gerber, S. A., Rush, J., & Gygi, S. P. (2006). A probability-based approach for high-throughput protein phosphorylation analysis and site localization. In Nature Biotechnology (Vol. 24, Issue 10, pp. 1285–1292). Springer Science and Business Media LLC. https://doi.org/10.1038/nbt1240

Johnson, J. L., Yaron, T. M., Huntsman, E. M., Kerelsky, A., Song, J., Regev, A., Lin, T.-Y., Liberatore, K., Cizin, D. M., Cohen, B. M., Vasan, N., Ma, Y., Krismer, K., Robles, J. T., van de Kooij, B., van Vlimmeren, A. E., Andrée-Busch, N., Käufer, N. F., Dorovkov, M. V., … Cantley, L. C. (2023). An atlas of substrate specificities for the human serine/threonine kinome. In Nature (Vol. 613, Issue 7945, pp. 759–766). Springer Science and Business Media LLC. https://doi.org/10.1038/s41586-022-05575-3

Eddins, D. J., Yang, J., Kosters, A., Giacalone, V. D., Pechuan-Jorge, X., Chandler, J. D., Eum, J., Babcock, B. R., Dobosh, B. S., Hernández, M. R., Abdulkhader, F., Collins, G. L., Orlova, D. Y., Ramonell, R. P., Sanz, I., Moussion, C., Eun-Hyung Lee, F., Tirouvanziam, R. M., & Ghosn, E. E. B. (2023). Transcriptional reprogramming of infiltrating neutrophils drives lung pathology in severe COVID-19 despite low viral load. In Blood Advances (Vol. 7, Issue 5, pp. 778–799). American Society of Hematology. https://doi.org/10.1182/bloodadvances.2022008834

Ariss, M. M., Huang, L., Ding, X., Sheth, S., Levy, T., Fisher, J., Loebelenz, J., Arlotta, K., Dixon, K., Polakiewicz, R., Kuchroo, V. K., & Beausoleil, S. A. (2024). InTraSeq: A Multimodal Assay that Uncovers New Single-Cell Biology and Regulatory Mechanisms. Cold Spring Harbor Laboratory. https://doi.org/10.1101/2024.09.19.613947

Agrawal, A., Balcı, H., Hanspers, K., Coort, S. L., Martens, M., Slenter, D. N., Ehrhart, F., Digles, D., Waagmeester, A., Wassink, I., Abbassi-Daloii, T., Lopes, E. N., Iyer, A., Acosta, J. M., Willighagen, L. G., Nishida, K., Riutta, A., Basaric, H., Evelo, C. T., … Pico, A. R. (2024). WikiPathways 2024: next generation pathway database. In Nucleic Acids Research (Vol. 52, Issue D1, pp. D679–D689). Oxford University Press (OUP). https://doi.org/10.1093/nar/gkad960

Liberzon, A., Subramanian, A., Pinchback, R., Thorvaldsdóttir, H., Tamayo, P., & Mesirov, J. P. (2011). Molecular signatures database (MSigDB) 3.0. In Bioinformatics (Vol. 27, Issue 12, pp. 1739–1740). Oxford University Press (OUP). https://doi.org/10.1093/bioinformatics/btr260

Browaeys, R., Saelens, W., & Saeys, Y. (2019). NicheNet: modeling intercellular communication by linking ligands to target genes. In Nature Methods (Vol. 17, Issue 2, pp. 159–162). Springer Science and Business Media LLC. https://doi.org/10.1038/s41592-019-0667-5

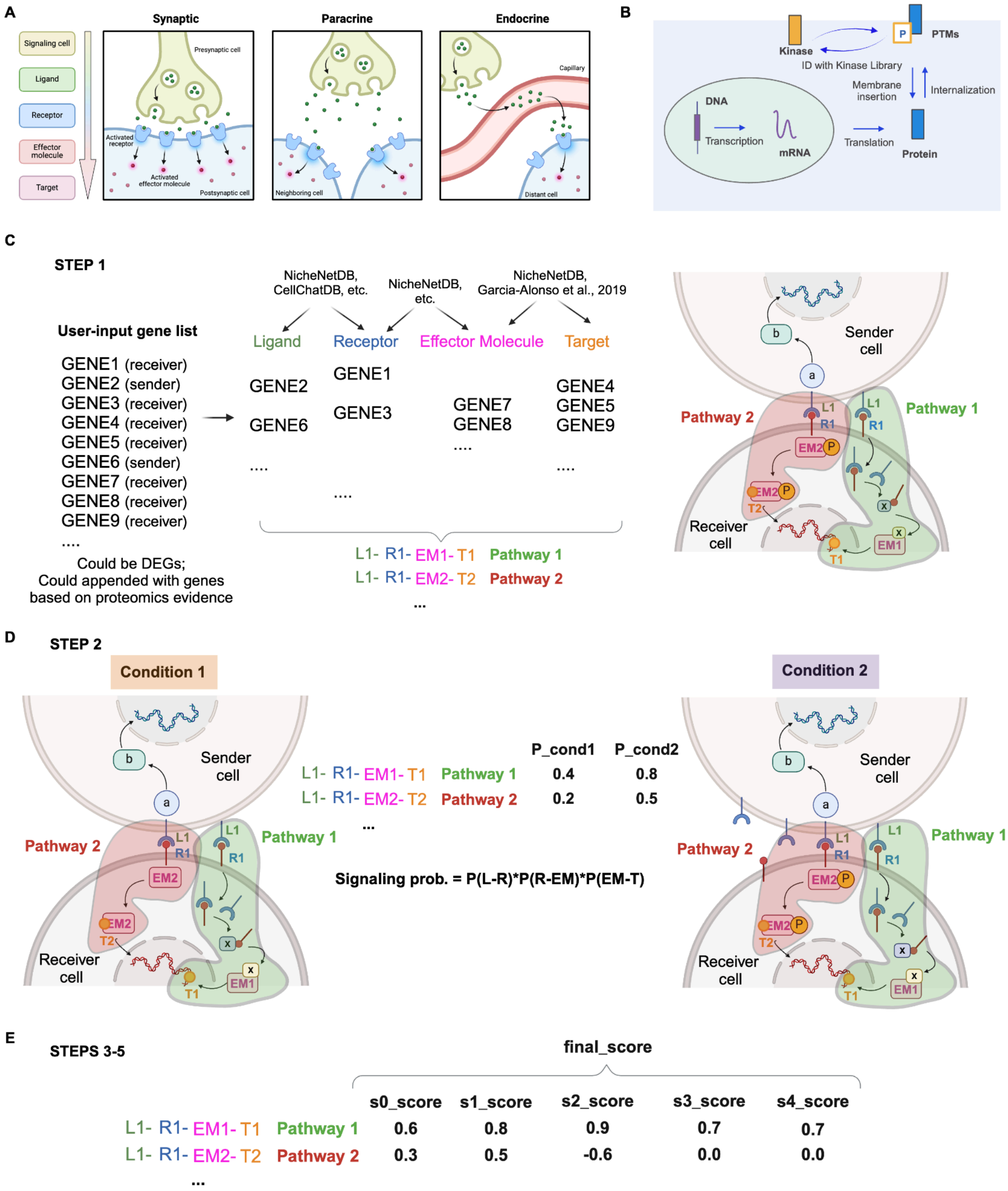

Supplementary Figure 1. A cartoon illustration depicting the steps of the Incytr algorithm. A. Since L-R-EM-T pathways are constructed using RNA expression data, the inference captures not only synaptic cell-cell communication but also accommodates paracrine and endocrine interactions. **B.** Incytr's cell signaling pathway inference is supported by evidence gathered from transcription, translation, and protein phosphorylation processes. **C-E.** Incytr pathway inference steps.

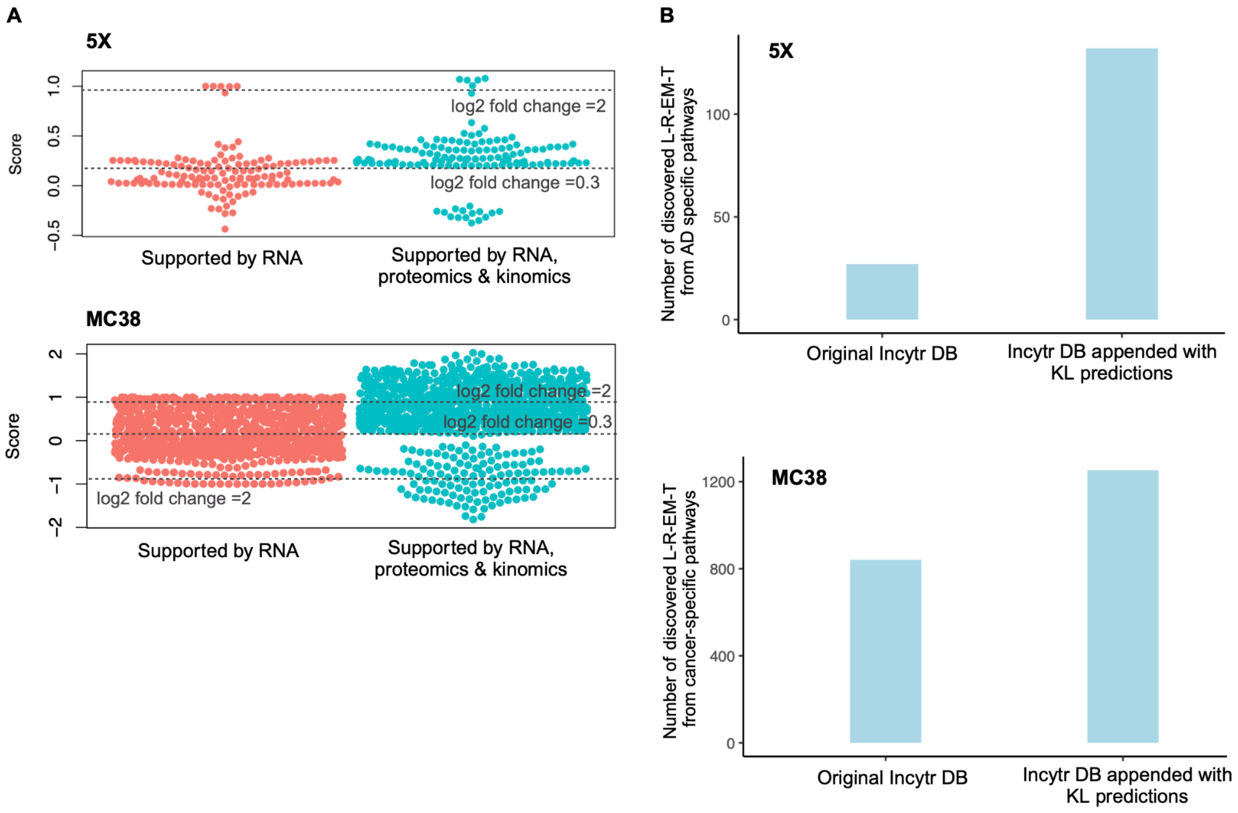

Supplementary Figure 2. Proteomics and kinomics data enable the reliable rediscovery of known pathways in Alzheimer's disease and cancer. A. Proteomics and kinomics data provide additional evidence, improving the scoring of pathways that might otherwise (based solely on mRNA data) be considered non-differential between WT and 5XFAD mice in the 5XFAD mouse model, and non-significant in MC38 mouse cancer model. Each point represents an L-R-EM-T pathway mapped to Alzheimer's disease (AD) or cancer-specific pathways (Focal Adhesion, VEGFR, Cancer Pathways) on WikiPathways. **B.** Appending the Incytr database with PPI predictions from the Kinase Library (KL) enables the discovery of over four times more L-R-EM-T pathways mapped to AD-specific pathways and over one-and-a-half times more L-R-EM-T pathways mapped to cancer-specific pathways on WikiPathways. This approach strengthens the evidence for the presence of these pathways in the given biological system.

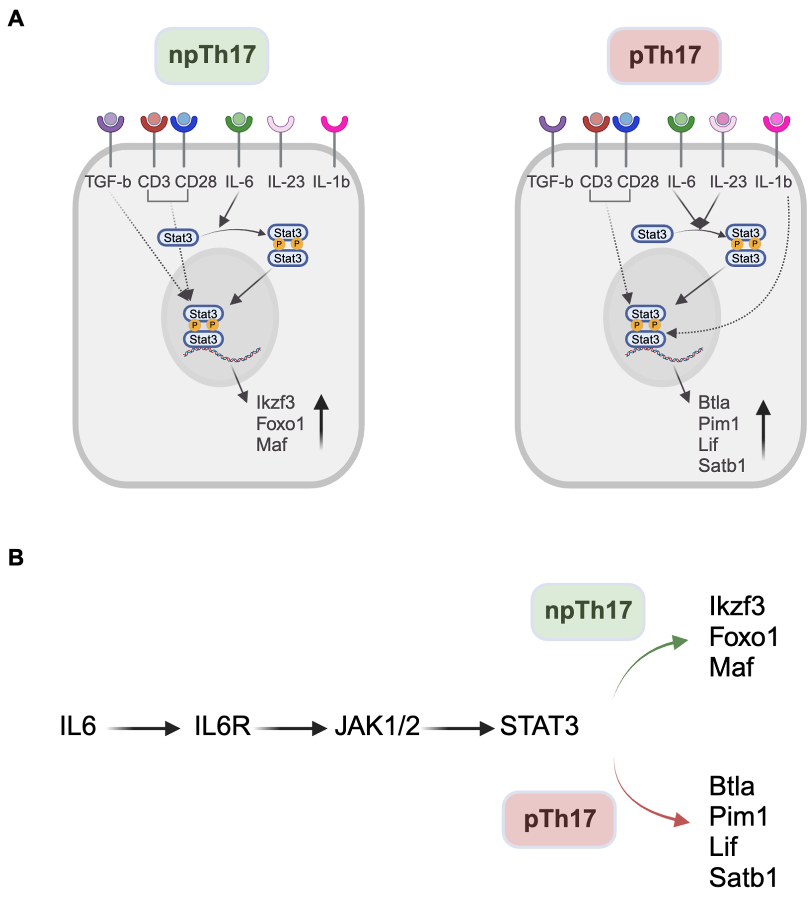

Supplementary Figure 3. Validation of Incytr in discovering functionally meaningful pathways. A. Graphical representation of non-pathogenic (npTh17; anti-CD3 + anti-CD28 + IL-6 + TGFβ) and pathogenic (pTh17; anti-CD3 + anti-CD28 + IL-6 + IL-1β + IL-23) stimulation conditions applied to naïve CD4+ T cells, as described [Ariss et al., 2024], and the role of Stat3 in differentiation. **B.** Signaling pathways identified by Incytr 24 hours post-stimulation. Artificial cell cluster X was added to the scRNAseq data with two cells per condition, expressing exclusively Il6 and Il6ra (at an expression level of the maximum expression of any gene in the real data). Incytr was run with X as the sender group.

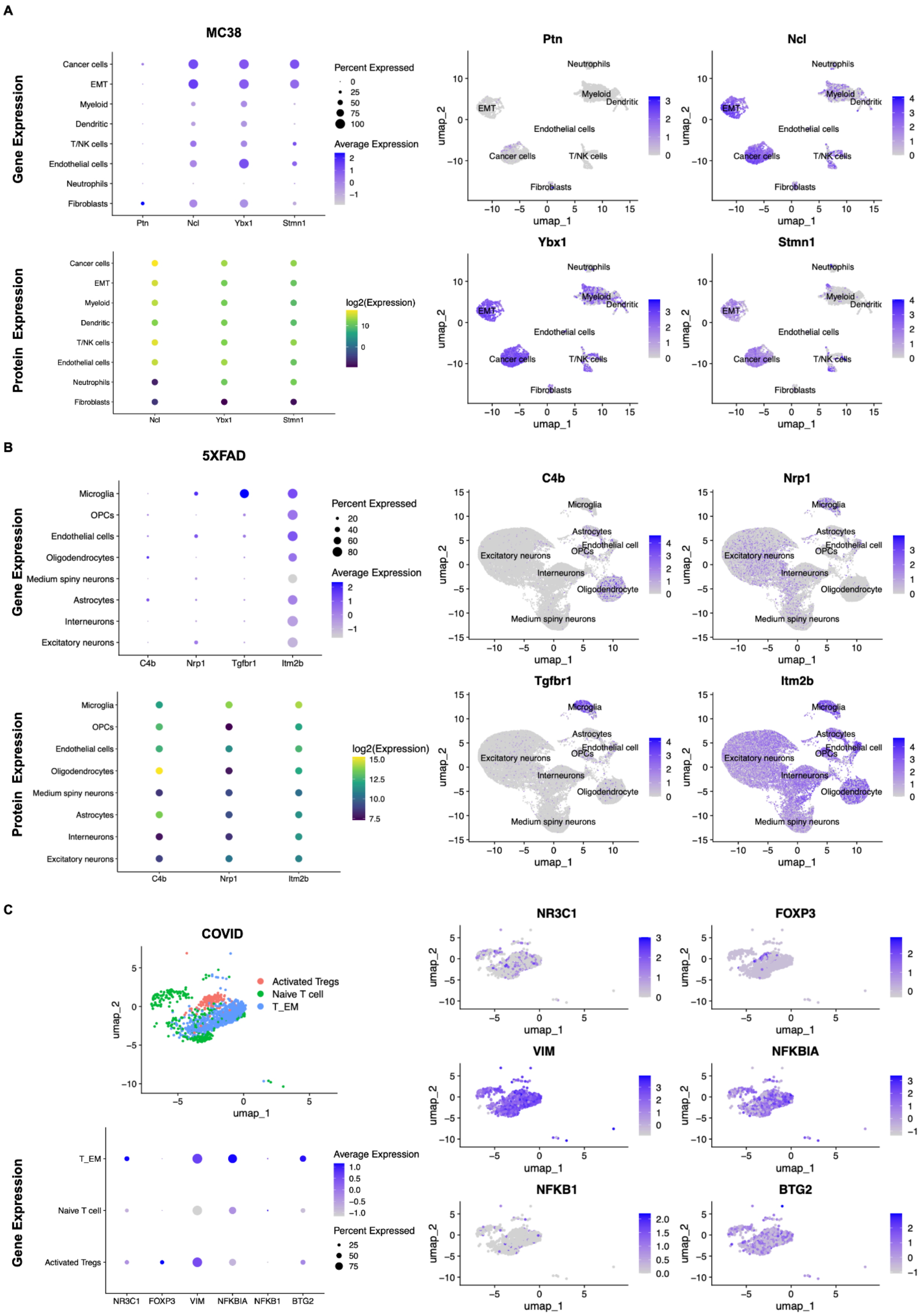
A
MC38
Gene Expression
Protein Expression
Cancer cells
EMT
Myeloid
Dendritic
T/NK cells
Endothelial cells
Neutrophils
Fibroblasts
Ptn
Ncl
Ybx1
Stmn1
Percent Expressed
Average Expression
log2(Expression)
umap_1
umap_2
B
5XFAD
Microglia
OPCs
Endothelial cells
Oligodendrocytes
Medium spiny neurons
Astrocytes
Interneurons
Excitatory neurons
C4b
Nrp1
Tgfbr1
Itm2b
Endothelial cell
Oligodendrocyte
C
COVID
Activated Tregs
Naive T cell
T_EM
NR3C1
FOXP3
VIM
NFKBIA
NFKB1
BTG2

Supplementary Figure 4. Gene and protein expression patterns for representative Incytr-inferred signaling pathways in the MC38 (A), 5XFAD (B), and primary patient cohort in COVID-19 (C) datasets.

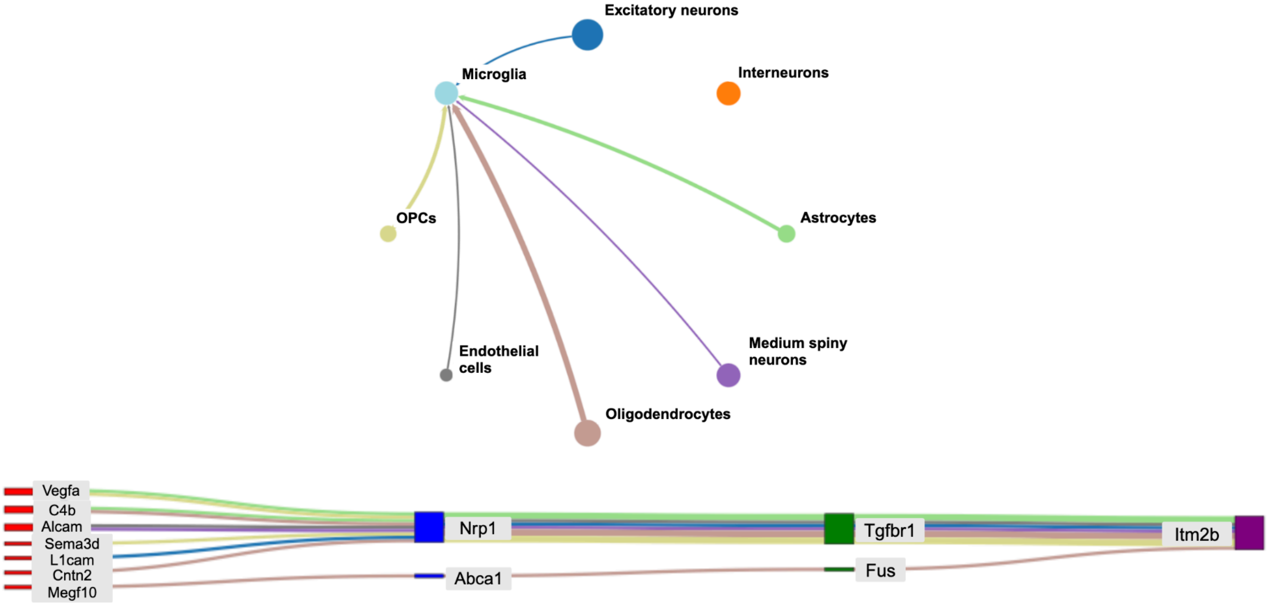

Supplementary Figure 5. Incytr-identified pathways showing differential expression (log2 fold change > 0.5 for T-PDS and PDS scores) in 5XFAD versus WT mice, involving the Itm2b gene in microglial cells.

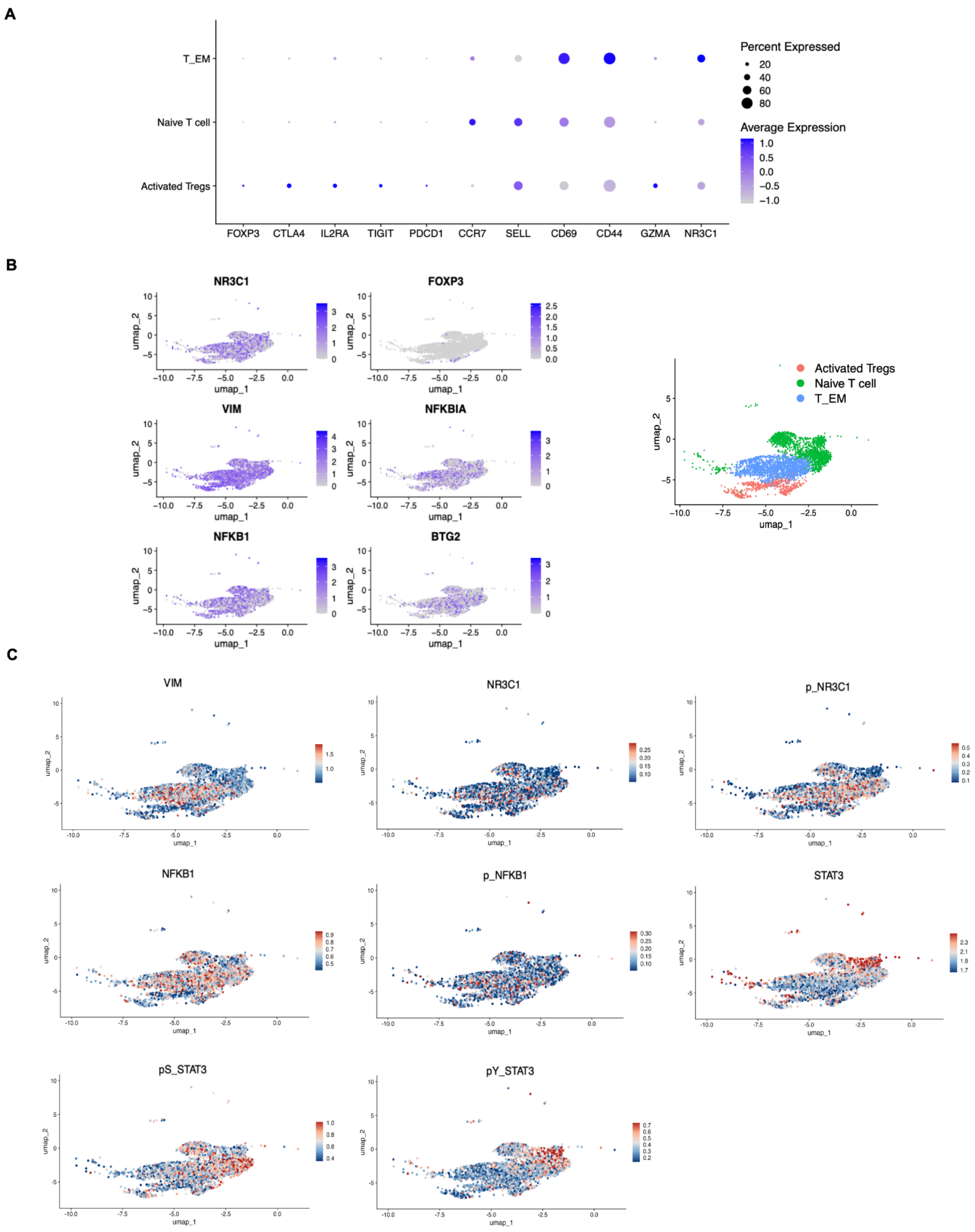

Supplementary Figure 6. Protein expression patterns in the second cohort of COVID-19 patients. A. Subclustering of the T cell population in this cohort aligns with T cell phenotyping from the first cohort shown in Figure 5B. **B.** mRNA expression profiles of the selected genes. **C.** InTraSeq results for selected proteins.

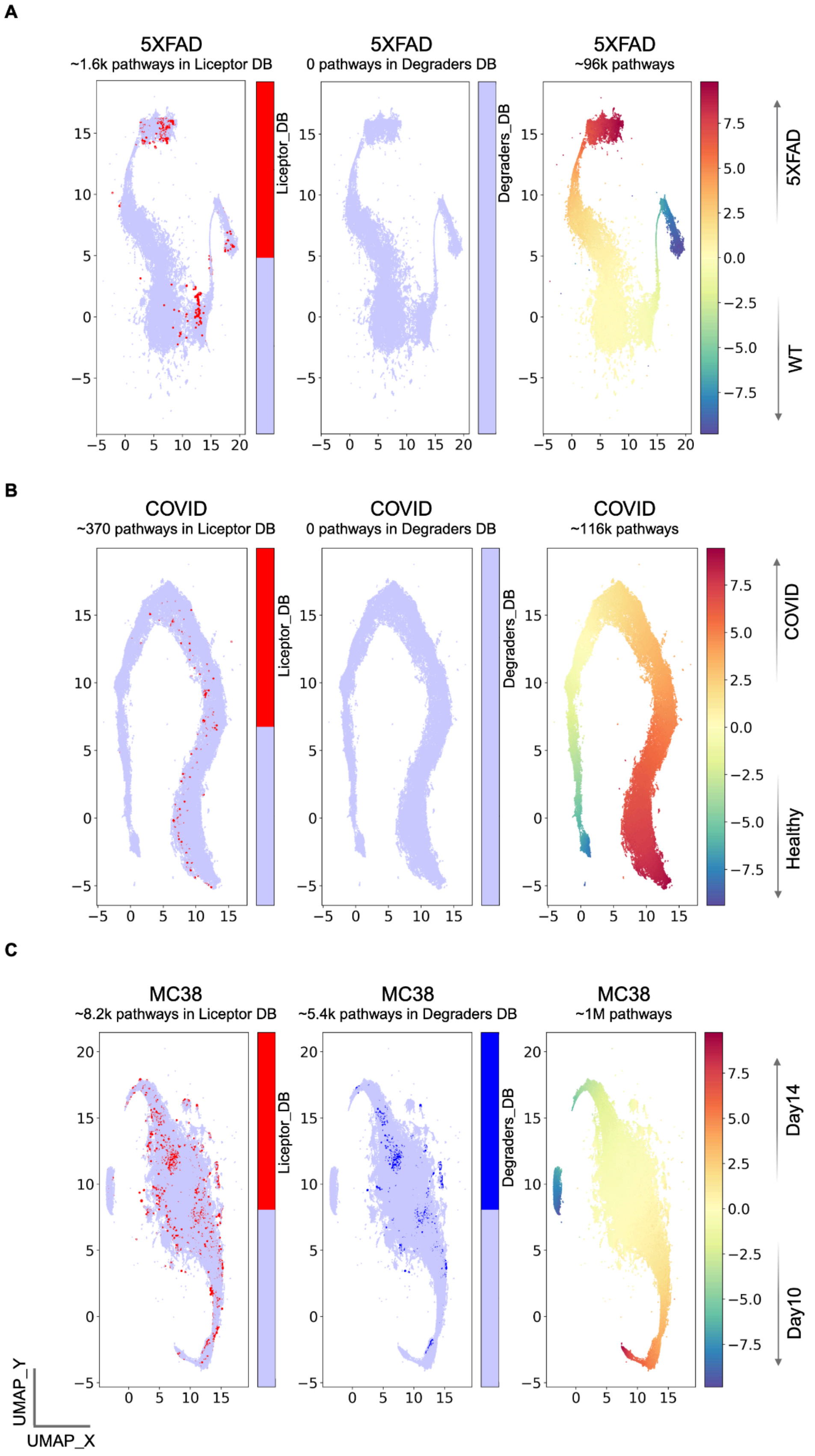
A
5XFAD
~1.6k pathways in Liceptor DB
Liceptor_DB
5XFAD
0 pathways in Degraders DB
Degraders_DB
5XFAD
~96k pathways
5XFAD
WT
B
COVID
~370 pathways in Liceptor DB
Liceptor_DB
COVID
0 pathways in Degraders DB
Degraders_DB
COVID
~116k pathways
COVID
Healthy
C
MC38
~8.2k pathways in Liceptor DB
Liceptor_DB
MC38
~5.4k pathways in Degraders DB
Degraders_DB
MC38
~1M pathways
Day14
Day10
UMAP_Y
UMAP_X

Supplementary Figure 7. The number of L-R-EM-T pathways annotated in the Liceptor and Degraders databases. Signaling pathways containing at least one molecule (L, R, EM, or T) annotated in the Liceptor or Degraders databases are highlighted in red or blue, respectively, in the 5XFAD (**A**), COVID (**B**), and MC38 (**C**) datasets.

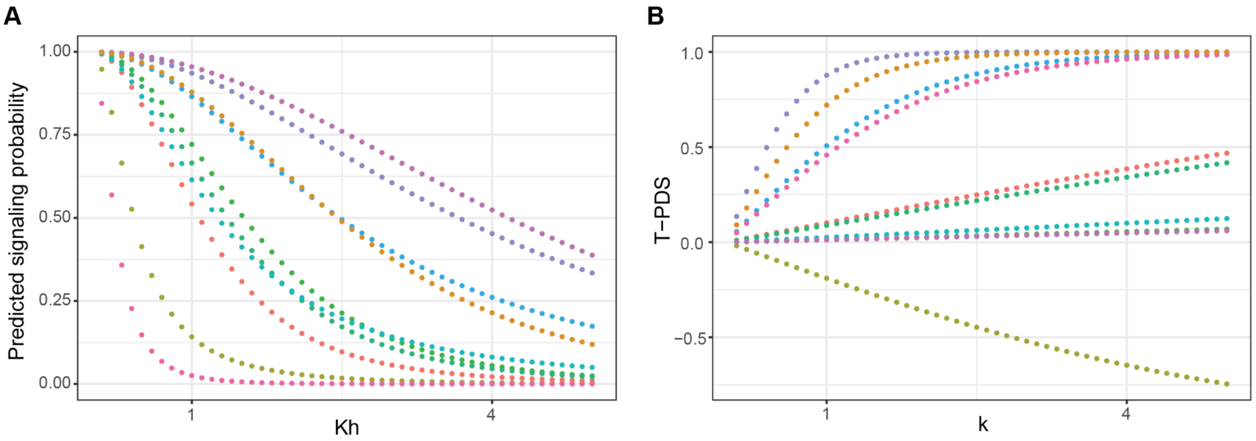

Supplementary Figure 8. The parameter perturbations of the signaling probability prediction and T-PDS calculation, respectively. A. Predicted signaling probabilities with different Kh values, showing that changing the value of Kh from 0.1 to 5 (with step size 0.1) does not change the order of signaling probabilities of the L-T pathways. Dot plot showing the predicted signaling probabilities of the synthetic data, where each gene has a random expression value between 0.1 to 5. **B.** Calculated T-PDS with different *k* values, showing that changing the value of *k* from 0.1 to 5 (with step size 0.1) does not change the order of T-PDS of the L-T pathways. Dot plot showing the calculated T-PDS of the synthetic data, where the signaling probabilities of each L-T pathway in two conditions are randomly picked between 0.1 and 1.

Concept	Definition	References
effector molecule (EM)	intermediate signal transmission molecule	New
target	molecule/gene that is regulated by the effector molecule	[Browaeys et al., 2019]
ligand-target signaling pathway	signaling pathway that starts from the ligand to the target molecule/gene	[Browaeys et al., 2019]
ligand-target signaling pathway (L-T pathway)	pathway that has the "ligand-receptor-EM-target" structure	New
signaling-involved kinase (SiK)	kinase which itself and its substrate are both the receptor/EM/target of an L-T pathway	New
signaling-related kinase (SrK)	kinase which is not SiK but predicted by KL to modify the substrate (receptor/EM/target of an L-T pathway)	New
adjusted fold-change value (aFC value)	adjusted log2 fold change to turn down the significant change made by two small numbers	New
exclusiveness index (EI)	the index that quantifies how exclusive a gene is expressed in the cell group	New
SiK-score	the score that quantifies how much a SiK contributes to the L-T pathway expression	New
transcriptomics-based pathway differential score (T-PDS)	the score is based on the analysis using the transcriptomics data and quantifies how an L-T pathway is differentially expressed between conditions	New
proteomics-based pathway differential score (P-PDS)	the score is based on the analysis using the proteomics data and quantifies how an L-T pathway is differentially expressed between conditions	New
phosphorylation-based pathway differential score (Ph-PDS)	the score is based on the analysis using the phosphorylation data and quantifies how an L-T pathway is differentially expressed between conditions	New
multi-modal differential	the score that quantifies how an L-T pathway	New

score	is differentially expressed between conditions based on the T-PDS, P-PDS, and Ph-PDS	
kinase-based pathway differential score (K-PDS)	the score that quantifies how much the identified SiKs contribute to the L-T pathway expression	New
pathway differential score (PDS)	the score that quantifies how an L-T pathway is differential between the conditions (includes T-PDS, P-PDS, Ph-PDS, and K-PDS)	New

Supplementary Table 1. A list of terminologies and concepts used in Incytr.

	Ligand-Receptor	Receptor-EM	EM-Target
Human	6869	3757666	4678245
Mouse	6707	3717790	4441402

Supplementary Table 2. The number of interactions recorded in the Incytr-DB.

Supplementary Data 1. Benchmarking table comparing the ability of GSEA, exFINDER, and Incytr to rediscover known cancer-specific, 5XFAD model-specific, and COVID-specific pathways.

Supplementary Data 2. A list of references supporting the primary pairwise L-R, R-EM, and EM-T interactions presented in this paper (Figures 3-5).

Supplementary Data 3. Output of the Incytr analysis on the 5XFAD dataset, focusing on interactions involving Apoe, App, Trem2, and Lrp1.
